# Overexpression of the maize transcription factor *ZmVQ52* accelerates leaf senescence in *Arabidopsis*

**DOI:** 10.1371/journal.pone.0221949

**Published:** 2019-08-30

**Authors:** Tingting Yu, Xuefeng Lu, Yang Bai, Xiupeng Mei, Zhifeng Guo, Chaoxian Liu, Yilin Cai

**Affiliations:** Maize Research Institute, Key Laboratory of Biotechnology and Crop Quality Improvement, College of Agronomy and Biotechnology, Southwest University, Chongqing, China; Institute of Genetics and Developmental Biology Chinese Academy of Sciences, CHINA

## Abstract

Leaf senescence plays an important role in the improvement of maize kernel yields. However, the underlying regulatory mechanisms of leaf senescence in maize are largely unknown. We isolated *ZmVQ52* and studied the function of *ZmVQ52* which encoded, a VQ family transcription factor. *ZmVQ52* is constitutively expressed in maize tissues, and mainly expressed in the leaf; it is located in the nucleus of maize protoplasts. Four WRKY family proteins—ZmWRKY20, ZmWRKY36, ZmWRKY50, and ZmWRKY71—were identified as interacting with *ZmVQ52*. The overexpression of *ZmVQ52* in *Arabidopsis* accelerated premature leaf senescence. The leaf of the *ZmVQ52*-overexpression line showed a lower chlorophyll content and higher senescence rate than the WT. A number of leaf senescence regulating genes were up-regulated in the *ZmVQ52*-overexpression line. Additionally, hormone treatments revealed that the leaf of the *ZmVQ52*-overexpressed line was more sensitive to salicylic acid (SA) and jasmonic acid (JA), and had an enhanced tolerance to abscisic acid (ABA). Moreover, a transcriptome analysis of the *ZmVQ52*-overexpression line revealed that *ZmVQ52* is mainly involved in the circadian pathway and photosynthetic pathways.

## Introduction

Leaf senescence plays a critical role in plant fitness and productivity [1]. The highly organized leaf senescence process provides a mechanism for mobilizing the nutrients that accumulate through photosynthesis and nutrient uptake to newly developing leaves or seeds [2]. And the onset and process of leaf senescence are influenced by various internal signals and environmental factors [3]. In the past decades, multiple layers of leaf senescence regulation have been revealed, including transcription factor (TF)-mediated regulation, chromatin-mediated regulation, post-transcriptional regulation, and post-translational regulation [4]. In addition, recently, light signaling and circadian clock were also demonstrated to participate in leaf senescence regulation [5, 6]. As they represent one of the most important layers of leaf senescence regulation, a number of TFs have been reported in recent years. For example, the TFs MYC2/3/4, bHLH03/13/14/17, WRKY57, JA ZIM domain TFs JAZ4, JAZ7 and JAZ8 are involved in leaf senescence-associated jasmonic acid (JA) signaling [7]. The TFs ANAC046, ANAC016, ANAC072 and ANAC029 activate genes that participate in chlorophyll catabolism [8–11]. In addition, EIN3 and ORE1/ANAC092 regulate ethylene-mediated chlorophyll degradation during leaf senescence in *Arabidopsis* [12].

Although a series of TFs have been identified in recent years, further characterization of TFs is necessary to gain more comprehensive insights into the global TF-mediated regulation of leaf senescence. Recently, several studies have uncovered the reciprocal interaction between circadian clock and leaf senescence in plant systems. For example, *TOC1* is necessary to integrate age-related information and regulate circadian periods [6]. The *PRR9*, a core circadian component, acts as a key regulator of leaf senescence via positive regulation of *ORE1* through a feed-forward pathway [1]. The *CCA1* directly activates *GLK2* and suppresses *ORE1* expression to counteract leaf senescence [13]. However, it is still uncertain how the leaf senescence process is associated with changes in the circadian system. Thus, it is necessary to uncover new cross nodes of leaf senescence and the circadian clock.

The VQ family genes, containing a unique and conserved VQ (FxxxVQxxTG) motif, respond to various environmental signals and play diverse roles in plant defense, growth, and development [14]. For example, in *Arabidopsis*, VQ14 regulates endosperm growth and seed size [15]. VQ23, VQ16 and VQ21 are required for the plant’s defense response [16–18], and VQ29 is a negative transcriptional regulator of the light-mediated inhibition of hypocotyl elongation [15]. In total, 61 VQ genes were identified in maize, among which *ZmVQ52*, without introns, is 576 bp in length and is located on chromosome 9 [15]. However, the function of *ZmVQ52* has not been reported before. Moreover, the reciprocal interaction between *ZmVQ52* and early leaf senescence in maize systems has not been documented.

In this study, we isolated the *ZmVQ52* gene, analyzed its expression pattern and subcellular localization to molecularly characterize the *ZmVQ52*. Furthermore, we overexpressed this gene in *Arabidopsis* to analyze the function of *ZmVQ52*. Finally, we selected the interacting proteins and conducted a transcriptome analysis of the *ZmVQ52-*overexpression line to elucidate the function of *ZmVQ52*. This study will lay a good foundation for elucidating the maize leaf senescence mechanism which, in turn, will, benefit maize breeding.

## Materials and methods

### Plant materials and experiment

The maize B73 inbred line was used in this experiment. For gene cloning, the total RNA of B73 was extracted to synthesize cDNA. For expression pattern analysis, the root, stem, leaf, tassel (1–2 cm) and ear (1–2 cm) of B73 were sampled for RNA extraction. The maize plants were grown at 25–30°C/16-18°C (day/night temperatures) under a light intensity of 220–260 mmol m^-2^ s^-1^ provided with a 14-h light/8-h dark cycle in a greenhouse under ~65% relative humidity.

The *Arabidopsis* ecotype Col-0 and two overexpression lines, OE-4 and OE-5, were used in the experiment. The seeds were surface-sterilized and sown on MS plates, and stratified for three days at 4°C. The plants were grown in an environmentally controlled growth room at 22°C with a16-h-light/8-h-dark cycle.

### Vector construction and transgenic plant generation

For constitutive overexpression of *ZmVQ52*, the *ZmVQ52* ORF was PCR amplified with cDNA of B73 and subsequently cloned into the binary vector pCAMBIA3301. The primers VQ52-F and VQ52-R are listed in the S1 Table. For the construction of transgenic plants, the vector CaMV35S::ZmVQ52 was transformed into *Arabidopsis* ecotype Col-0 using the floral dip method mediated by the *Agrobacterium tumefaciens* strain EHA105. Transgenic plants were screened with Glufosinate (5 mg/L, Sigma) and confirmed by PCR analysis.

### Subcellular localization

The full-length coding sequence (CDS) of the ZmVQ52 protein without the stop code was PCR amplified with the primers sVQ52-F and sVQ52-R, listed in S1 Table. The CDS was then cloned into the vector pCAMBIA1300-GFP, under the control of the CaMV 35S promoter, to construct a ZmVQ52-GFP fusion protein. Protoplasts were isolated from the maize seedlings of using a modification of a previously described method [19]. The mCherry nuclear location marker mCherry-N was used as a control to co-transform with ZmVQ52-GFP [20]. The GFP protein co-transformed with mCherry-N and used as a control. To allow the introduced genes to be expressed, protoplasts were incubated in the dark at room temperature for 16–24 h. Then confocal microscopy images were taken using a LSM800 confocal laser scanning microscope (Carl Zeiss, NY, USA).

### Measurement of chlorophyll content, senescence rate

Chlorophyll was extracted from individual leaves by heating in 95% ethanol at 80°C. Chlorophyll concentration was calculated based on fresh weight of the leaf tissue [21]. For the senescence rate calculation, leaves with 50% of leaf area yellowed were counted as senesced, and the senescence rate was the proportion of senesced leaves to total number of leaves [1].

### BIFC

The vector pDOE-03 was used for bimolecular fluorescence complementation (BiFC) assay. Using the B73 cDNAs as template, each ORF of the five genes—*ZmWRKY20*, *ZmWRKY36*, *ZmWRKY50*, *ZmWRKY71* and *ZmVQ52*—was PCR amplified using the primers listed in S1 Table. The ORF of *ZmVQ52* was inserted into the MCS1 site and the ORFs of WRKY genes without the stop codon were inserted into MCS3. The resulting plasmid contained a recombinant gene, which was confirmed by sequencing. The mCherry nuclear location marker mCherry-N was used as a control to co-transform with the constructs. The maize protoplast transformation method was the same as above.

### Treatments with salicylate (SA), jasmonic (JA) and abscisic acids (ABA)

SA, JA, and ABA were purchased from Sigma-Aldrich (St Louis, MO, USA). Plants were sprayed with 1 mM SA solution, and leaf samples were taken at different time-points after the treatment. For the JA treatment, plants were sprayed with 100 μM methyl jasmonate [16]. For the ABA treatment, 5-day-old plants were transplanted into MS medium with 0, 1 or 2 μM ABA for one week before root length measurement, using method modified from one previously reported [22].

### Transcriptome analysis

For the “OE-5 vs WT” transcriptome analysis, the four-week-old leaf tissue was sampled for RNA extraction. The purity of RNA was checked using a NanoPhotometer spectrophotometer (Implen, CA, USA). The concentration of RNA was measured using the Qubit RNA Assay Kit with a Qubit 2.0 Fluorometer (Life Technologies, CA, USA). cDNA libraries were constructed and RNAseq was performed by using the Illumina HiSeq 2000 platform. A differential expression analysis of samples was performed using the DEGSeq R package 1.20.0. The P values were adjusted using the Benjamini and Hochberg method. The differentially expressed genes (DEGs) with an adjusted P < 0.05 were employed for gene ontology (GO) enrichment analysis using the online tool WEGO (Web Gene Ontology Annotation Plot, http://wego.genomics.org.cn). The KOBAS2.0 software (http://kobas.cbi.pku.edu.cn/home-do) was used to test the statistical enrichment of the DEGs in the KEGG pathways.

### qRT-PCR analysis

First-strand cDNA was generated from total RNA using the RevertAid First Strand cDNA Synthesis Kit following the manufacturer’s protocol (Thermo Scientific). qRT-PCR was performed on a Bio-Rad platform (CFX96) using a SYBR Green detection chemistry kit (SYBR® Premix ExTaq^™^, TaKaRa). Each 13 μL mixture contained 6.25 μL of SYBR Green Supermix (TaKaRa), 1.0 μL of cDNA, 0.375 μL of each primer, and distilled water. The program used for qRT-PCR was as follows: 95°C for 30 s; followed by 35 cycles of denaturation at 95°C for 5 s and annealing at 62°C for 30 s. The housekeeping genes *ZmActin3* and *AtActin2* were each used as an internal control. All primers used for qRT-PCR are given in S2 Table. The 2-ΔCT method was used to estimate the fold change. The data were analyzed using the Bio-Rad CFX Manager software. Three biological replicates with three technical replicates were used for each reaction.

## Results

### *ZmVQ52* is mainly expressed in maize leaf

**Fig 1:**
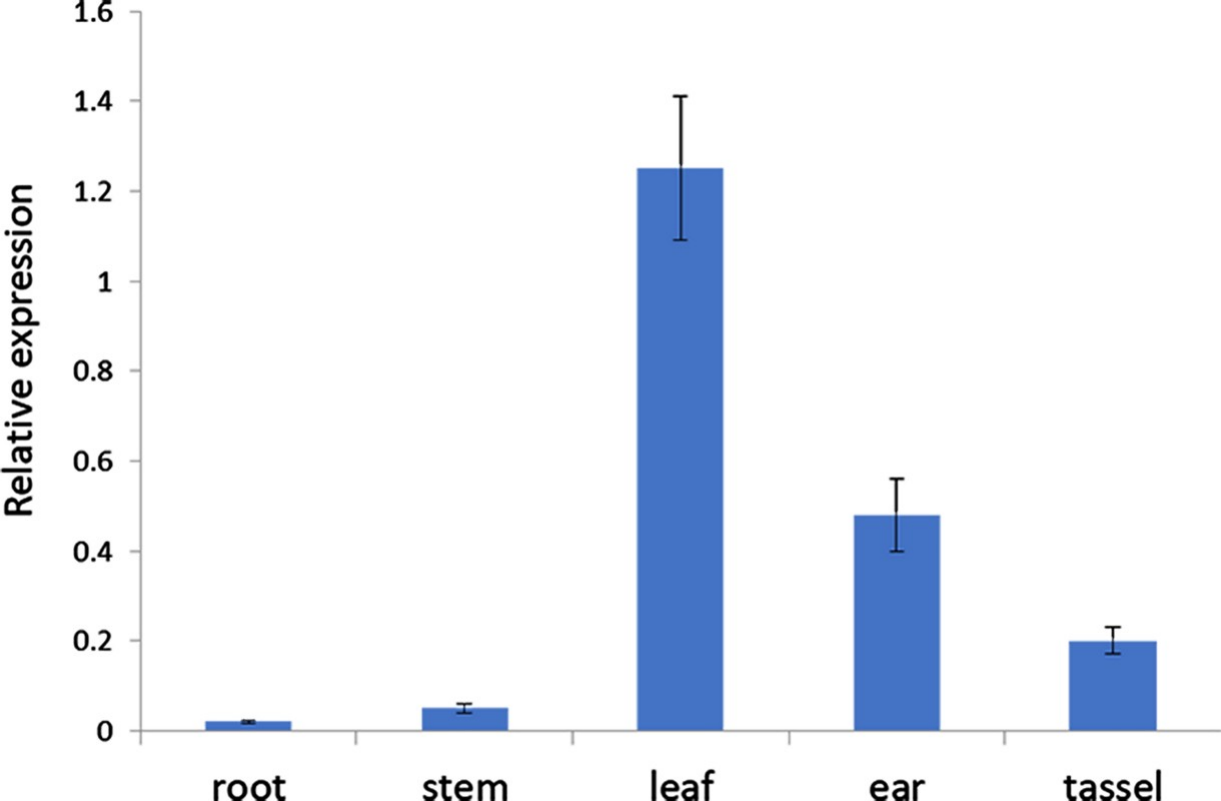
Expression pattern of *ZmVQ52* in root, stem, leaf, ear (1–2 cm) and tassel (1–2 cm) of maize B73 inbred line. Three replicates were used in the experiment. Values are the means ± SD (n = 3). *ZmActin3* expression was used as the internal control.

In order to analyze the expression pattern of *ZmVQ52*, qRT-PCR analysis was conducted in maize root, stem, leaf, ear (1–2 cm) and tassel (1–2 cm) from the maize B73 inbred line. The results of expressing *ZmVQ52* in all tissues indicated that it was constitutively expressed. The gene was mainly expressed in the leaf, with low expression levels in other tissues, revealing that it was only expressed in certain tissues (Fig 1).

### *ZmVQ52* is located in the nucleus

**Fig 2:**
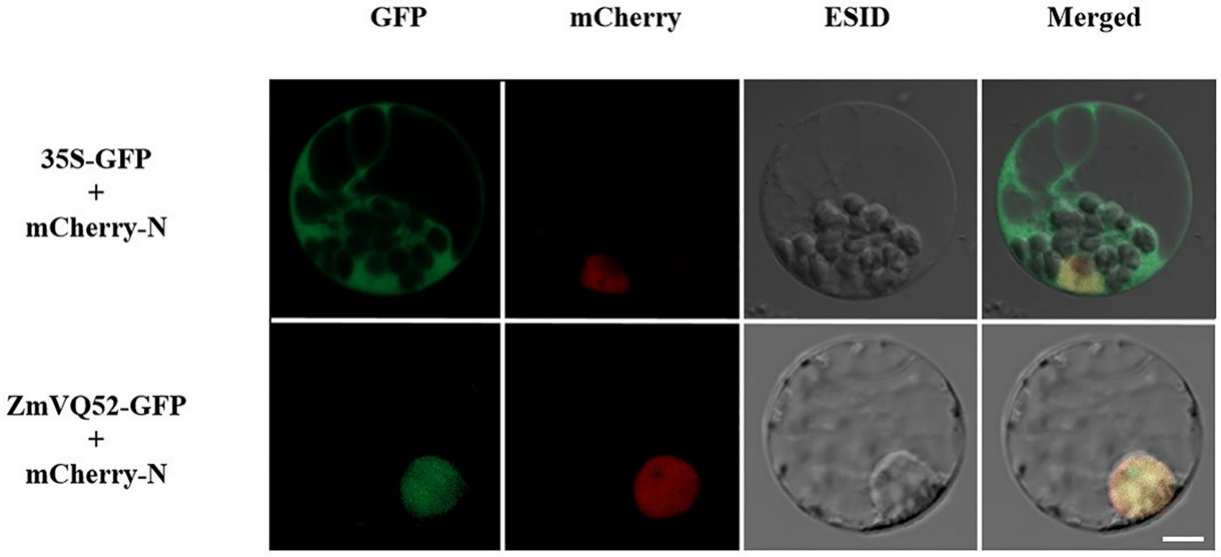
Subcellular localization of *ZmVQ52*. Confocal scanning images show *ZmVQ52* co-localized with a mCherry-labeled nucleus marker (mCherry-N). The vector pCAMBIA1300, in which sGFP was regulated the CaMV 35S promoter, served as the control. Bars = 10 μm.

To determine the functional localization of ZmVQ52, the full length of *ZmVQ52* without the stop codon was fused with green fluorescent protein (GFP) and driven by a constitutive Cauliflower mosaic virus (CaMV) 35S promoter. The vectors for the expression of the ZmVQ52-GFP fusion protein and the mCherry nuclear location marker (mCherry-N) were co-transformed into maize protoplasts, and the GFP protein co-transformed with the mCherry-N co-transformed was used as a control. The results showed that the ZmVQ52-GFP fusion protein and the mCherry-N both co-localized in the nucleus. Green florescence was observed throughout the protoplasts that expressed the GFP protein and mCherry-N. These results revealed that ZmVQ52 localized in the nucleus, implying that ZmVQ52 functions as a transcriptional regulator (Fig 2).

### *ZmVQ52* interacted with *ZmWRKY20, ZmWRKY36, ZmWRKY50* and *ZmWRKY71*

**Fig 3:**
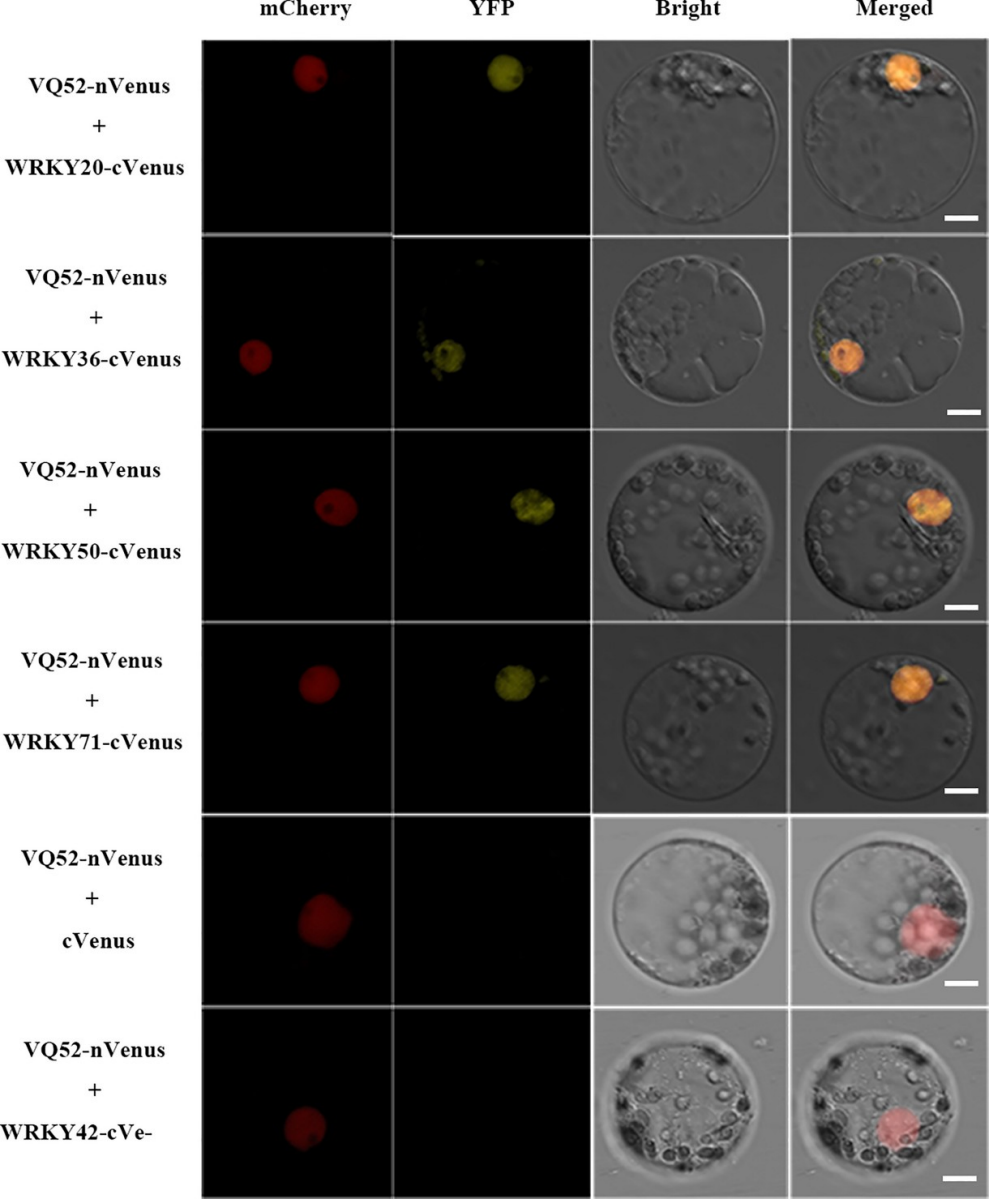
Identification of *ZmVQ52*-interacting protein by BiFC. BiFC in maize Mo17 protoplasts showed the interaction between ZmVQ52 and *ZmWRKY20, ZmWRKY36, ZmWRKY50* and ZmWRKY71. *ZmVQ52* was fused with the N terminus (YN) of YFP. The four WRKY proteins were each fused with the C terminus (YC) of YFP. The fusion proteins were delivered into maize protoplasts and visualized using a confocal microscope. Bars = 10 μm.

The majority of VQ family proteins studied so far can interact with WRKY transcription factors [23]. According to a previous study, WRKY3, WRKY4, WRKY20, WRKY23, WRKY25, WRKY33, WRKY34, WRKY10, WRKY24, WRKY51, and WRKY75 are the most important WRKY proteins to interact with VQ proteins in *Arabidopsis* [24]. Hence, the orthologous genes of these WRKY proteins in maize were used for interaction protein selection. To identify the interaction protein of ZmVQ52, the coding sequences of the maize WRKY genes were cloned for BiFC vector construction. The interactions between ZmVQ52 and the WRKY family protein were investigated using the maize protoplast expression system. The results showed that ZmWRKY20, ZmWRKY36, ZmWRKY50 and ZmWRKY71 co-localized with ZmVQ52 in the nucleus (Fig 3). Therefore, these four WRKY family proteins interacted with ZmVQ52.

### The overexpression of *ZmVQ52* accelerates age-dependent leaf senescence in *Arabidopsis*

**Fig 4:**
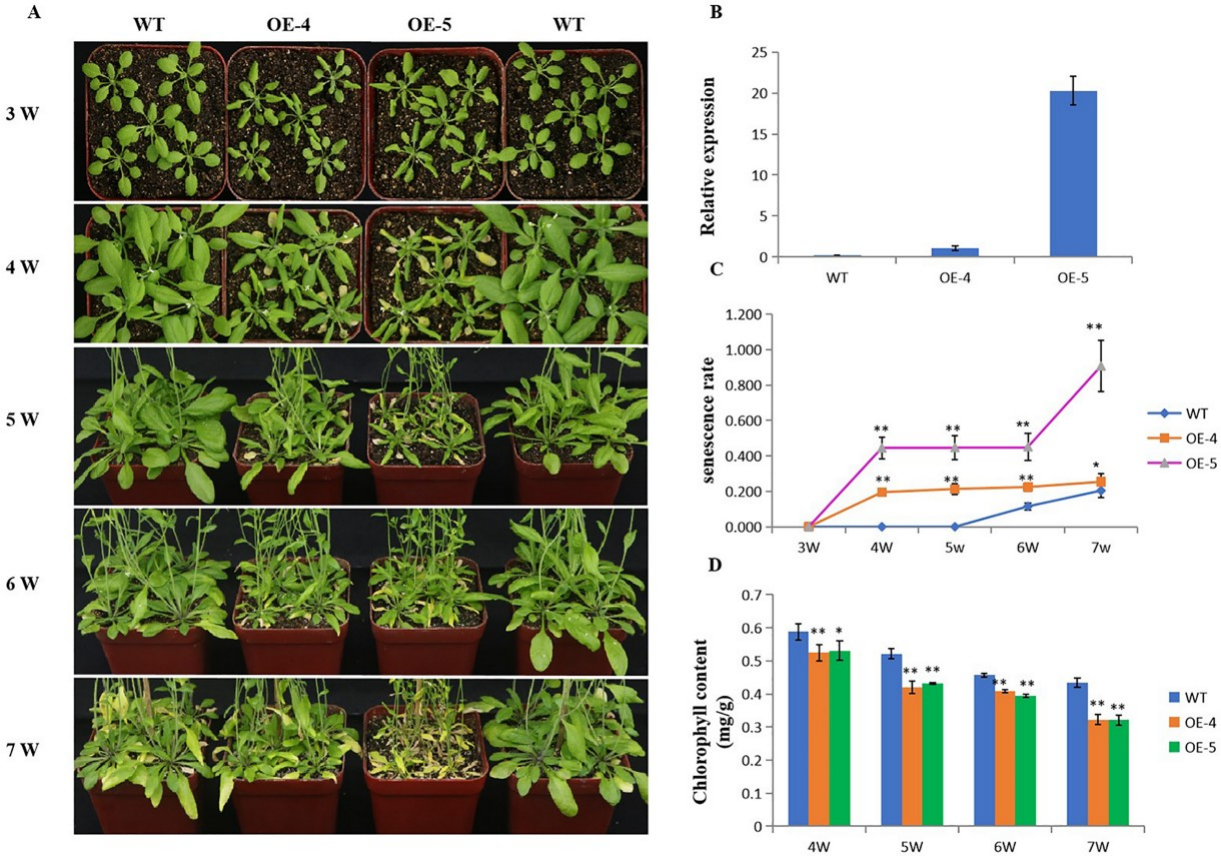
Age-dependent leaf senescence phenotypes of constitutive *ZmVQ52-*overexpression lines. (A) Growth of the WT and *ZmVQ52* *Arabidopsis* transgenic plants OE-4 and OE-5 at different time points. (B) The expression levels of *ZmVQ52* in the WT, OE-4 and OE-5. (C) The senescence rate of leaves at different time points. (D) The chlorophyll content of leaf at different time points. Three replicates were used in the experiment. Values are the means ± SD (n = 10). The comparisons were made using Student’s t test. *, **Significant differences at P = 0.05 and 0.01, respectively.

To explore the function of ZmVQ52 in leaf senescence, Arabidopsis transgenic lines OE-4 and OE-5 overexpressing *ZmVQ52* were generated for phenotype analysis. Senescence symptoms were analyzed during age-dependent leaf senescence. OE-4 and OE-5 exhibited premature leaf senescence and dwarfing with partly curled leaves compared to the WT at different time points (Fig 4A). Moreover, the severity of the phenotypes correlated with the expression levels of ZmVQ52 (Fig 4B). The leaf senescence ratio was significantly higher in the *ZmVQ52*-overexpression lines from 4 to 7 weeks as compared to the WT (Fig 4C). Additionally, the leaf chlorophyll content of OE-4 and OE-5 was significantly decreased from week 4 to 7 (Fig 4D). The results indicated that the overexpression of *ZmVQ52* accelerated leaf senescence in *Arabidopsis*.

### The overexpression of *ZmVQ52* enhanced expression of leaf senescence responsive genes

**Fig 5:**
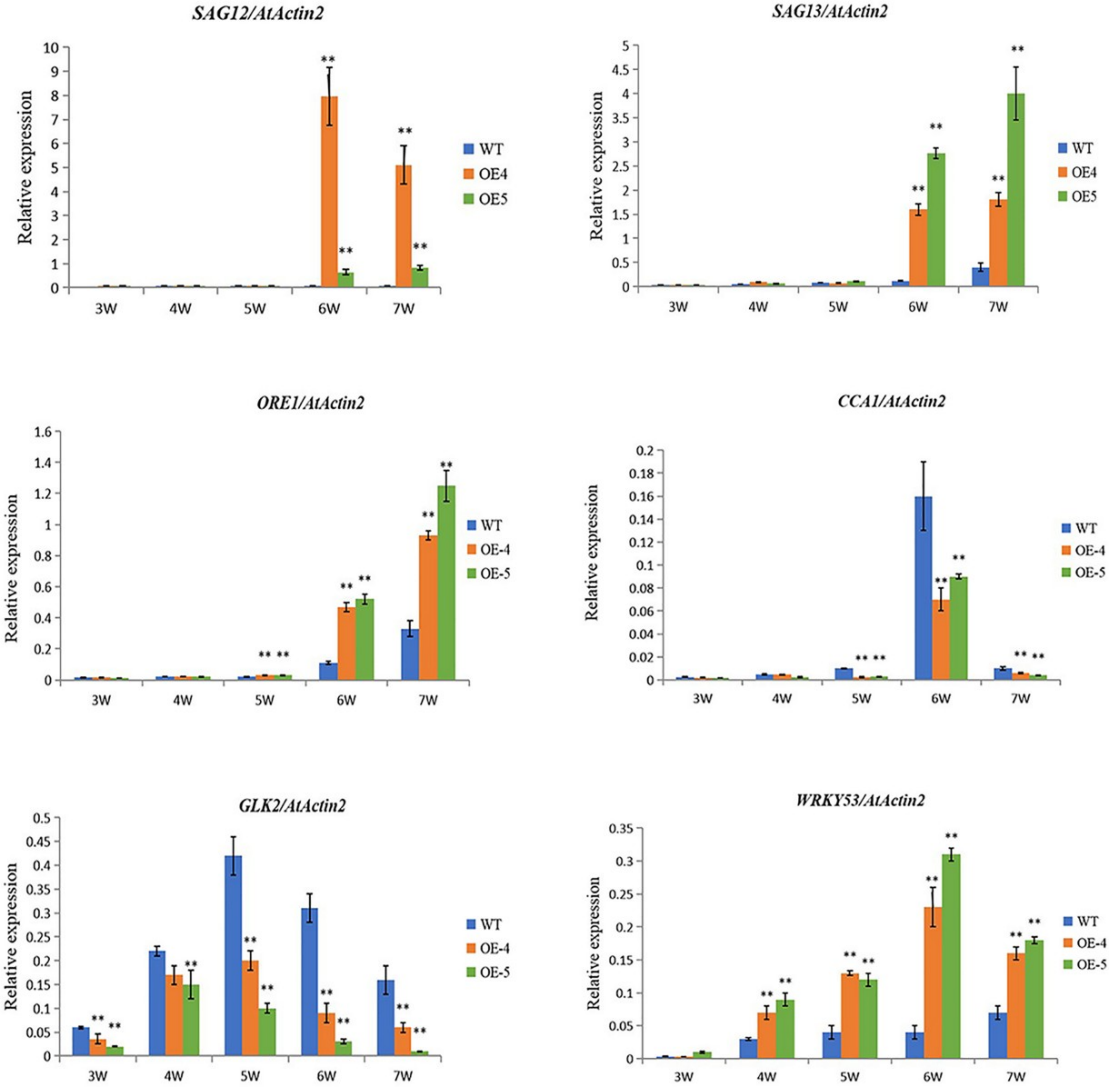
Age-dependent changes in gene expression. The expression levels of *SAG12*, *SAG13*, *ORE1*, *CCA1*, *GLK2* and *WRKY53* under normal conditions at different points. Three replicates were used in the experiment. Values are the means ± SD (n = 3). The comparisons were made using Student’s t test. *, **Significant differences at P = 0.05 and 0.01, respectively. *AtActin2* expression was used as the internal control.

To gain a deeper insight into the function of ZmVQ52 in regulating leaf senescence, the expression patterns of several leaf senescence responsive genes were analyzed. ORE1, WRKY53, CCA1 and GLK2 all play important roles in leaf senescence. CCA1 inhibits leaf senescence through directly activating GLK2 and suppressing ORE1 expression [13]. The expression levels of WRKY53 and ORE1 were significantly higher in the *ZmVQ52*-overexpression line, while those of CCA1 and GLK2 were significantly lower in the *ZmVQ52*-overexpression line than in the WT (Fig 5). In order to confirm the senescence phenotype of the ZmVQ52-overexpression lines, we analyzed the expression levels of two senescence marker genes, SAG12 and SAG13 [25], The result was that the expression levels of OE-4 and OE-5 lines were significantly higher than that of the WT (Fig 5), which is consistent with the senescence phenotype of leaves. The investigation of the expression pattern of these genes revealed that ZmVQ52 may function as a node in the crosstalk between circadian rhythm and leaf senescence.

### The overexpression lines of *ZmVQ52* enhanced sensitivity to JA and SA, tolerance to ABA

**Fig 6:**
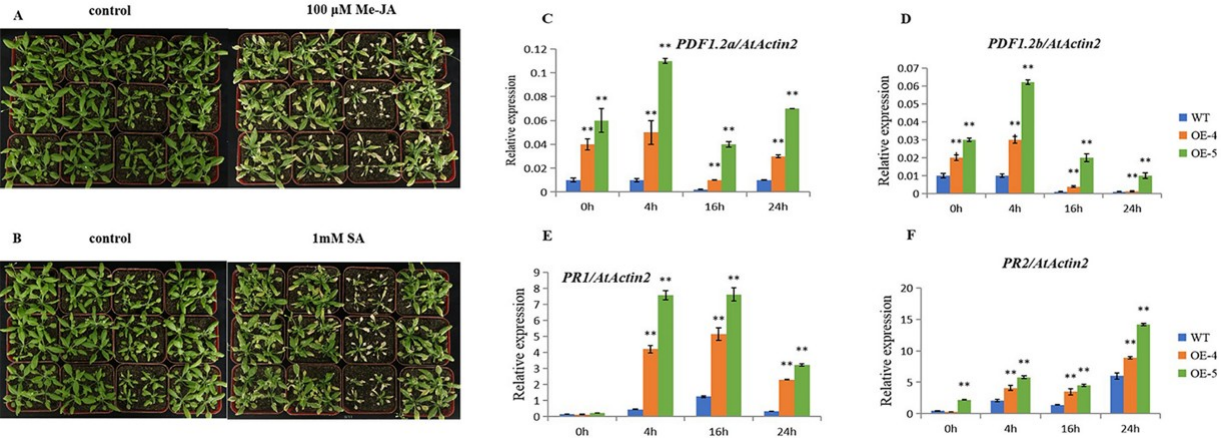
Phenotypes and relative expression levels of genes in response to JA and SA treatments. (A) The growth condition of *Arabidopsis* after JA (100μM) treatment. (B) The growth condition of *Arabidopsis* after SA(1mM) treatment. RNA was extracted from 4-week-old leaves taken at different time points after JA and SA treatments. The relative expression levels of PDF1.2a (C), PDF1.2b (D) PR1 (E) and PR2 (F) were determined by qRT-PCR analysis. In addition, *PDF1*.*2a* and *PDF1*.*2b* were the JA signaling marker genes involved in senescence [26]. *PR1* was the SA signaling marker gene involved in senescence [25]. Three replicates were used in the experiment. Values are the means ± SD (n = 3). The comparisons were made using Student’s t test. *, **Significant differences at P = 0.05 and 0.01, respectively. *AtActin2* expression was used as the internal control.

In order to investigate whether the *ZmVQ52* is involved in the JA or SA pathways, we sprayed four-week-old plants with JA (100 μM) and SA (1 mM) solutions. Then the leaves were sampled at 0 h, 4 h, 16 h, and 24 h. One week after the JA treatment, the OE-4 and OE-5 plants showed yellow and brownish dry leaves, while most WT leaves remained green (Fig 6A). The JA-responsive genes *PDF1*.*2a* and *PDF1*.*2b* were significantly upregulated in OE-4 and OE-5 at different time points, including 0 h (Fig 6C and 6D). This result suggested that the overexpression of *ZmVQ52* upregulated the expression of JA-responsive genes, regardless of whether JA was present. One week after the SA treatment, the OE-4 and OE-5 plants also showed yellow and brownish dry leaves, while most WT leaves remained green (Fig 6B). The SA-responsive genes *PR1* and *PR2* were upregulated in OE-4 and OE-5 at 4 h, 16 h and 24 h (Fig 6E and 6F). This result implied that overexpression of *ZmVQ52* upregulated the expression of SA-responsive genes in response to SA treatment. Taken together, *ZmVQ52* also participated in the JA and SA pathways.

**Fig 7:**
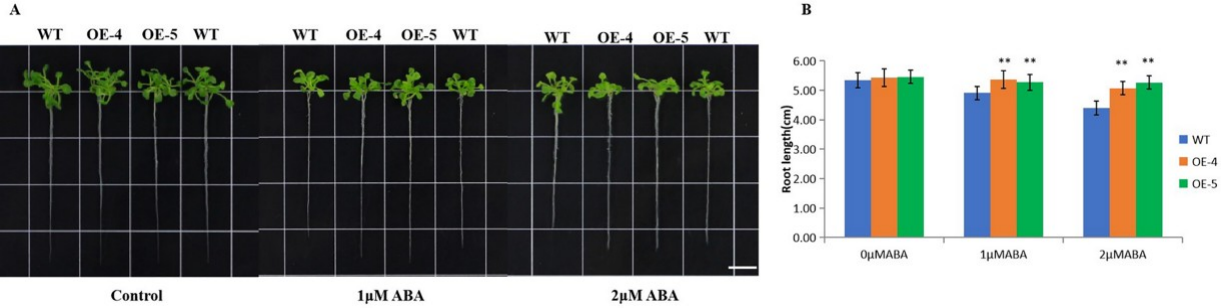
Phenotypes and root length in response to ABA treatment. (A) Photographs of the WT, OE-4 and OE-5 in 1 μM ABA and 2 μM ABA MS media for 7 days. Bar = 1 cm. (B) Primary root length of the WT, OE-4 and OE-5 in 1 μM ABA and 2 μM ABA MS media for 7 days. Three replicates were used in the experiment. Values are the means ± SD (n = 20). The comparisons were made using Student’s t test. *, **Significant differences at P = 0.05 and 0.01, respectively.

To assess the role of *ZmVQ52* in response to the ABA treatment, the primary root length of the *Arabidopsis* plant was also measured. There was no difference in the root length of plants grown in control medium. The root length of OE-4 and OE-5 were significantly longer than WT in MS medium with 1 μM ABA and 2 μM ABA (Fig 7). The result indicated that the overexpression of *ZmVQ52* enhanced tolerance to ABA treatment.

### *ZmVQ52* regulated leaf senescence mainly through photosynthesis and circadian rhythm pathways

To further analyze the *ZmVQ52*-mediated leaf senescence regulation mechanism, the transcriptome analysis of OE-5 vs WT was conducted. Two cutoffs were used to identify the DEGs, log2|FC|≧1, FDR and q-value ≤ 0.01. The results indicated that 2863 genes were differentially expressed, among which 1106 were significantly upregulated and 1757 were significantly downregulated.

**Fig 8:**
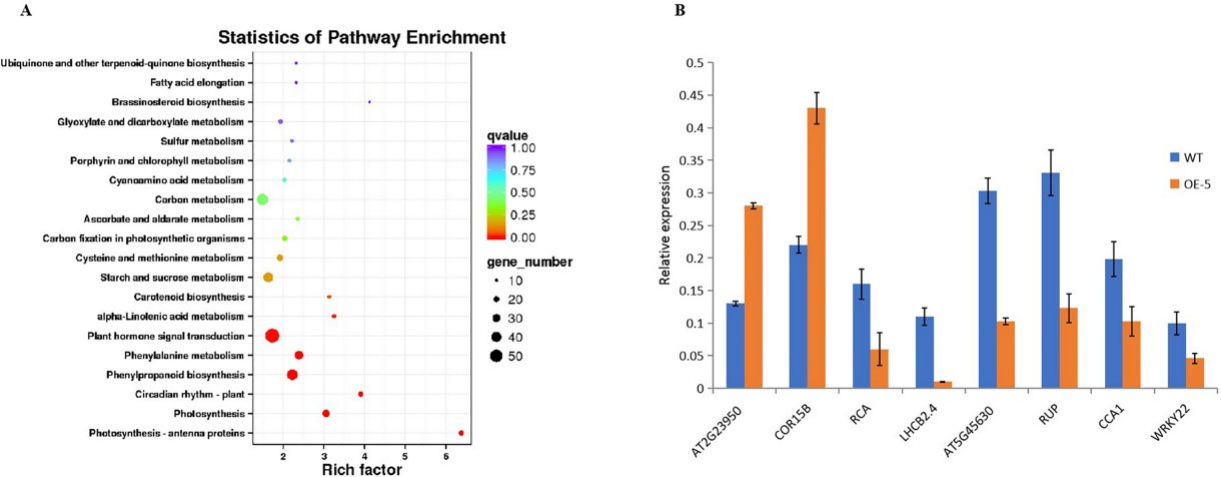
Statistics of pathway enrichment and qRT-PCR verification in transcriptome analysis. (A) The statistics of pathway enrichment showing the ratio of the proportion of genes annotated to a pathway in these differential genes to the proportion of genes in all genes annotated to that pathway. (B) qRT-PCR verification of transcriptome analysis. The eight genes are differentially expressed genes obtained by transcriptome data analysis and belong to different pathways. Among them, *AT2G23950*, *AT5G45630*, *COR15B*, *WRKY22*, and *RCA* are genes in the leaf senescence pathway, and *CCA1* is a gene in the circadian rhythm-plant pathway, *RUP2* is a gene in the photosynthesis pathway, and *LBCB2*.*4* is a gene in the photosynthesis-antenna proteins pathway. Values are the means ± SD (n = 3). *AtActin2* expression was used as the internal control.

According to the KEGG analysis, photosynthesis-antenna proteins, photosynthesis, and plant circadian rhythms were the most significantly enriched pathways (Fig 8A). We further analyzed the DEGs and found 11 genes directly related to leaf senescence, including two upregulated genes and nine downregulated genes (Table 1). Five genes encoding photosynthesis-antenna proteins were significantly downregulated, including four genes encoding photosystem II light harvesting complex proteins and one gene encoding chlorophyll A/B-binding protein (Table 1). Three genes involved in the photosynthesis process were also differentially expressed. Moreover, 10 genes involved in circadian rhythm were significantly differentially expressed, with seven genes upregulated and three genes downregulated (Table 1). The result indicated that *ZmVQ52* accelerated leaf senescence mainly through photosynthesis-antenna proteins, photosynthesis and plant circadian rhythms pathways.

**Table 1:** The overrepresented genes up- or downregulated in the *ZmVQ52*-overexpression lines and probably involved in the leaf senescence process.

Fig	Gene name	WT_FPKM	OE5_FPKM	Log2FC	Description	Regulated
Leaf senescence						
AT1G66330	AAF	91.5277	17.54568	2.41	senescence-associated family protein	down
AT1G69490	NAP	13.7987	0.956336	3.82	NAC-like, activated by AP3/PI	down
AT1G76680	OPR1	51.52591	21.66465	1.28	12-oxophytodienoate reductase 1	down
AT2G42530	COR15B	2.22	297.522	6.91	cold regulated 15b	up
AT2G42540	COR15A	8.54	247.59602	4.77	cold-regulated 15a	up
AT3G11340	UGT76B1	8.59194	2.66049	1.71	UDP-Glycosyltransferase superfamily protein	down
AT4G01250	WRKY22	30.1383	3.96687	2.93	WRKY family transcription factor	down
AT4G16690	ATMES16	21.6442	4.96862	2.08	methyl esterase 16	down
AT5G01820	ATSR1	76.648	29.6106	1.39	serine/threonine protein kinase 1	down
AT5G13170	SAG29	1.46182	0.147418	2.82	senescence-associated gene 29	down
AT5G51720	NEET	612.505	153.329	1.85	2 iron, 2 sulfur cluster binding protein	down
Circadian rhythm—plant						
AT1G68050	ADO3	0	59.2403	10.17	flavin-binding, kelch repeat, f box 1	up
AT5G60100	PRR3	0.1333	49.4305	8.17	pseudo-response regulator 3	up
AT5G24470	APRR5	0.2195	27.3065	6.63	two-component response regulator-like protein	up
AT5G15840	CO	0.2898	5.62812	4.01	B-box type zinc finger protein with CCT domain-containing protein	up
AT5G61380	TOC1	5.99901	95.1724	3.98	CCT motif-containing responseregulator protein	up
AT2G25930	ELF3	7.686764	69.5184	3.05	hydroxyproline-rich glycoprotein family protein	up
AT1G22770	GI	3.8471	29.981	2.94	gigantea protein (GI)	up
AT1G01060	LHY	93.7311	0.2436	8.56	Homeodomain-like superfamily protein	down
AT2G46830	CCA1	123.9455	0.3195	8.49	circadian clock associated 1	down
AT2G46790	APRR9	21.2142	0.2110	6.34	pseudo-response regulator 9	down
Photosynthesis						
AT5G47080	CKB1	17.2985	40.6816	1.20	casein kinase II beta chain 1	up
AT5G23730	RUP2	23.9039	1.48625	3.94	Transducin/WD40 repeat-like superfamily protein	down
AT3G17609	HYH	83.2752	11.0364	2.91	HY5-homolog	down
Photosynthesis-antenna proteins						
AT3G27690	LHCB2.4	3129.73	65.692	5.62	photosystem II light harvesting complex protein 2.3	down
AT2G05100	LHCB2.1	6501.77	317.912	4.42	photosystem II light harvesting complex protein 2.1	down
AT2G05070	LHCB2.2	6300.04	334.556	4.30	photosystem II light harvesting complex protein 2.2	down
AT5G54270	LHCB3	9015.37	1147.61	3.04	light-harvesting chlorophyll B-binding protein 3	down
AT1G29920	CAB2	10978.9	974.889	3.58	chlorophyll A/B-binding protein 2	down

To verify the transcriptome data, eight genes were selected for qRT-PCR analysis (Fig 8B). The results showed that the trends of these genes were consistent with the transcriptome data. This result indicated the reliability and accuracy of transcriptome result.

## Discussion

In *Arabidopsis*, the functions and mechanisms of VQ family genes were better elucidated. For example, VQ14 regulated endosperm growth and seed size [15]; VQ23, VQ16 and VQ21 are required for the plant defense response [16–18]; VQ29 is a negative transcriptional regulator of the light-mediated inhibition of hypocotyl elongation [15]. In maize, although 61 VQ genes have been identified, their functions and mechanisms are largely unknown. In this study, we identified a novel maize VQ gene, *ZmVQ52*. It was mainly expressed in maize leaves and subcellularly located in the nucleus (Figs 1 and 2). These results lay a good foundation for studying the function and mechanism of *ZmVQ52*.

In order to study the function of *ZmVQ52*, the gene was transformed into *Arabidopsis*. Compared with the WT, the transgenic lines had higher expression levels of ZmVQ52, higher leaf senescence rates, and lower leaf chlorophyll contents (Fig 4). These results suggest that *ZmVQ52* accelerates leaf senescence in *Arabidopsis*. In maize breeding, it may delay senescence by down-regulating the expression of *ZmVQ52*.

To further confirm the function and elucidate the mechanism of *ZmVQ52* in regulating leaf senescence, we identified the interacting proteins, analyzed the expression of leaf senescence- responsive genes, and performed a transcriptome analysis of the transgenic line vs WT. A number of WRKY genes played crucial parts in leaf senescence. In *Arabidopsis*, WRKY22, WRKY45, WRKY53 and WRKY75 play positive roles in accelerating leaf senescence [27–30]. WRKY54, WRKY57, and WRKY70 function as negative regulators of leaf senescence [31, 32]. In rice, OsWRKY42 and OsWRKY23 function in enhancing leaf senescence [33, 34]. In this study, four WRKY family proteins were identified as the interaction proteins of ZmVQ52 by BiFC, namely, ZmWRKY20, ZmWRKY36, ZmWRKY50 and ZmWRKY71 (Fig 3). The orthologs of these four genes in *Arabidopsis* are *AtWRKY3*, *AtWRKY75*, *AtWRKY51*, and *AtWRKY23* [24]. In particular, AtWRKY75, the orthologous gene of ZmWRKY36, positively regulates leaf senescence in *Arabidopsis thaliana*, suggesting ZmWRKY36 may play an important role during leaf senescence in maize [30]. *AtWRKY3*, the orthologous gene of ZmWRKY20, plays a role in plant defense [35]. *AtWRKY51*, the orthologous gene of *ZmWRKY50*, also plays a role in SA- and JA-mediated defenses [36]. Among the four interaction proteins, only AtWRKY23 was differently expressed in transgenic line comparing with WT by transcriptome analysis. Based on gene function annotation, AtWRKY23 response to auxin in *Arabidopsis thaliana*. Therefore, we speculated that this gene may be involved in the progress of plant development. However, how AtWRKY23 regulates the leaf senescence needs to be further studied.

The overexpression of *ZmVQ52* upregulated the positive regulatory factors *ORE1* and *WRKY53* and downregulated the negative regulatory factors *CCA1* and *GLK2* in response to leaf senescence under normal conditions (Fig 5), and upregulated the JA-responsive genes *PDF1*.*2a* and *PDF1*.*2b* under JA treatment conditions and the SA-responsive genes *PR1* and *PR2* under SA treatment conditions (Fig 6C, 6D, 6E and 6F). Furthermore, the genes involved in the JA and SA pathways were also detected in the transcriptome analysis (Table 1). These results suggested the existence of an interconnection between *ZmVQ52* and the hormone signaling pathway during leaf senescence.

Transcriptome analysis of OE-5 vs WT showed that there were 11 leaf differentially expressed senescence-related genes (Table 1). Of these, two genes were upregulated and the other nine were downregulated. Of these genes, *AAF* is involved in redox homeostasis to regulate leaf senescence mediated by age and stress factors during *Arabidopsis* development [37]. *AtWRKY22* participates in the dark-induced senescence signal transduction pathway [28]. The SAG29 protein may serve as a molecular link that integrates environmental stress responses in the senescence process [38]. These genes were also differentially expressed in our study. These results further proved that *ZmVQ52* plays a role in leaf senescence. The transcriptome analysis also showed that 10 genes involved in circadian rhythm were overrepresented. In particular, the core clock component PRR9, acting as a positive leaf senescence regulator, was significantly downregulated, which suggests that PRR9 may function upstream of *ZmVQ52* in the leaf senescence regulatory pathway. The genes *ELF3*, *TOC1*, *PRR3*, *PRR5* and *GI* were significantly upregulated in OE-5, while *LHY* and *CCA1* were significantly downregulated (Table 1) in the transgenic line. This transcriptome analysis further confirms that *ZmVQ52* acts as a novel intersection between circadian systems and early leaf senescence.

## Supporting information

S1 Table Primers used for gene cloning in this study. (XLS)

S2 Table Primers used for qRT-PCR in this study. (XLS)
